# Proarrhythmic Remodeling of Calcium Homeostasis in Cardiac Disease; Implications for Diabetes and Obesity

**DOI:** 10.3389/fphys.2018.01517

**Published:** 2018-10-30

**Authors:** Shanna Hamilton, Dmitry Terentyev

**Affiliations:** ^1^Department of Medicine, The Warren Alpert Medical School of Brown University, Providence, RI, United States; ^2^Cardiovascular Research Center, Rhode Island Hospital, Providence, RI, United States

**Keywords:** Ca^2+^-dependent cardiac arrhythmia, diabetes, heart failure, L-type Ca^2+^ channels, Na^+^-Ca^2+^ exchanger type 1, ryanodine receptor type 2, sarco/endoplasmic reticulum Ca^2+^-ATPase type 2a

## Abstract

A rapid growth in the incidence of diabetes and obesity has transpired to a major heath issue and economic burden in the postindustrial world, with more than 29 million patients affected in the United States alone. Cardiovascular defects have been established as the leading cause of mortality and morbidity of diabetic patients. Over the last decade, significant progress has been made in delineating mechanisms responsible for the diminished cardiac contractile function and enhanced propensity for malignant cardiac arrhythmias characteristic of diabetic disease. Rhythmic cardiac contractility relies upon the precise interplay between several cellular Ca^2+^ transport protein complexes including plasmalemmal L-type Ca^2+^ channels (LTCC), Na^+^-Ca^2+^ exchanger (NCX1), Sarco/endoplasmic Reticulum (SR) Ca^2+^-ATPase (SERCa2a) and ryanodine receptors (RyR2s), the SR Ca^2+^ release channels. Here we provide an overview of changes in Ca^2+^ homeostasis in diabetic ventricular myocytes and discuss the therapeutic potential of targeting Ca^2+^ handling proteins in the prevention of diabetes-associated cardiomyopathy and arrhythmogenesis.

## Introduction

Heart failure (HF) and sudden cardiac death (SCD) due to malignant ventricular arrhythmias remain a major cause of mortality and morbidity in the developed world, in part due to alarming growth in the rates of obesity and diabetes (Benjamin et al., 2018). Diabetic patients have a two-fold increased risk for SCD and approximately 70 % suffer cardiovascular complications (Chugh et al., 2008; Laakso, 2008; Spooner, 2008; Bergner and Goldberger, 2010; Siscovick et al., 2010; Vasiliadis et al., 2014). Defective intracellular Ca^2+^ homeostasis has been established as a key contributor to diabetes-related cardiac dysfunction and enhanced arrhythmogenesis independent of coronary heart disease or hypertension (Belke and Dillmann, 2004; Aneja et al., 2008; Junttila et al., 2010; Pappone and Santinelli, 2010; Axelsen et al., 2015; Singh et al., 2018).

Diabetes is a chronic metabolic disorder characterized by hyperglycemia and reduced glucose utilization due to defective insulin secretion or action (American Diabetes Association, 2009). Type 1 diabetes is caused by the autoimmune destruction of pancreatic β-cells and a deficiency in insulin production. Linked with increasing rates of obesity, the more prevalent Type 2 diabetes is caused by cellular resistance to insulin and the failure of β-cells to compensate. In the diabetic heart, mitochondrial energetics are altered, with a switch of metabolic substrate from glucose to fatty acids (Boudina and Abel, 2010; Bertero and Maack, 2018). An imbalance between energy production and substrate utilization results in increased myocardial oxygen consumption and lipotoxicity, leading to mitochondrial dysfunction and reduced cardiac efficiency (Bhatti et al., 2017; Bertero and Maack, 2018). Altered mitochondrial energetics also drive changes in morphology and enhance the emission of reactive oxygen species (ROS), resulting in increased oxidative stress of the myocardium (Shen et al., 2004; Dabkowski et al., 2009; Joubert et al., 2018). It is well established that cardiovascular complications are common in both types of diabetes (Laakso, 2008; Bergner and Goldberger, 2010).

In the heart the process of excitation-contraction (EC) coupling drives cyclic changes in intracellular Ca^2+^ concentration [Ca^2+^], leading to rhythmic contraction and relaxation of cardiomyocytes in response to variable metabolic demand (Bers, 2002). Cardiac contractility is precisely regulated by ion channels and exchangers that maintain beat-to-beat Ca^2+^ concentrations in the steady state, whereby Ca^2+^ influx must equal Ca^2+^ efflux (Eisner D. et al., 2013). Depolarization of the sarcolemma and activation of voltage-dependent L-type Ca^2+^ channels (LTCCs) leads to Ca^2+^ influx into the cytosol. This small influx subsequently triggers a much larger Ca^2+^-induced Ca^2+^ release (CICR; Fabiato, 1985) from the sarcoplasmic reticulum (SR) Ca^2+^ stores through ryanodine receptors (RyR2s). The significant global increase in cytosolic [Ca^2+^], known as the Ca^2+^ transient, activates contractile machinery and leads to muscle contraction. For relaxation to then occur, Ca^2+^ must be sequestered and the intracellular [Ca^2+^] decreased. This is primarily by extrusion from the cytosol via the Na^+^/Ca^2+^ exchanger (NCX1), or resequestration into the SR via the sarco/endoplasmic reticulum-ATPase (SERCa2a).

Abnormal function of components of Ca^2+^ cycling machinery has been implicated in reduced contractility and proarrhythmic electrical instabilities in a variety of inherited and acquired cardiac diseases including HF and diabetic cardiomyopathy (Lagadic-Gossmann et al., 1996; Aneja et al., 2008; Jia et al., 2018). Reduction of SR Ca^2+^ release during systole contributes to diminished contraction, while enhancement of spontaneous Ca^2+^ release promotes early and delayed after-depolarizations (i.e., EADs and DADs respectively) of sarcolemma implicated in initiation of triggered activity in the heart (Landstrom et al., 2017). In addition to trigger for arrhythmia initiation, abnormal Ca^2+^ cycling contributes to arrhythmia substrate to maintain and perpetuate it via beat-to-beat alternations of electrical activity of the heart, i.e., alternans (Edwards and Blatter, 2014).

Despite many similarities in diabetes-related remodeling of Ca^2+^ homeostasis in comparison to that in HF, there are also some differences. This may also be complicated by the various animal models and species used to study the condition, highlighted in the review of King (2012). Models of Type 1 diabetes include chemically induced hyperglycemia by injection of streptozotocin (STZ) or alloxan (Szkudelski, 2001), as well as animals with genetically induced β-cell destruction (Mathews et al., 2002). Type 2 diabetes is modeled in both obese and non-obese animals. Genetically obese models include *ob/ob*, *db/db* and Zucker diabetic fatty (ZDF) hyperglycemic rodents, while obesity can also be induced by high fat diet (HFD) or high-sucrose diet (King and Bowe, 2016). Although larger animal models have been studied more recently (Xie et al., 2013; Zhang et al., 2017; Liang et al., 2018; Yang et al., 2018), most research investigating diabetes-related ventricular arrhythmias to date has been performed on rodents and remains limited. Conversely, functional alterations of Ca^2+^ handling proteins and EC coupling in HF have been extensively researched over several decades, in both small and large animal models as well as failing human cardiomyocytes (Hasenfuss et al., 1994; Studer et al., 1994; Schmidt et al., 1999; Louch et al., 2004; Sossalla et al., 2010; Crossman et al., 2011; Ottolia et al., 2013; Zima et al., 2014; Gorski et al., 2015; Høydal et al., 2018).

To place defective Ca^2+^ homeostasis in the context of our current understanding of EC coupling in cardiac disease, this review summarizes the changes and contribution of major cardiac Ca^2+^ handling proteins LTCC, RyR2, SERCa2a, and NCX1 to the reduced cardiac contractility observed in both HF and diabetes. We discuss the role of perturbed EC coupling in arrhythmogenesis in diabetes and the potential of targeting Ca^2+^ handling proteins as an anti-arrhythmic strategy.

## L-Type Ca^2+^ Channel

Ca^2+^ influx though voltage-dependent L-type Ca^2+^ channels (LTCC) during action potential initiates Ca^2+^ release from the sarcoplasmic reticulum (SR). The LTCC consists of the pore forming subunit α1c, and regulatory subunits α2/δ and β2 (Muralidharan et al., 2017). C-terminus associated calmodulin (CaM) confers Ca^2+^-dependent inactivation of the channel (Peterson et al., 1999; Zühlke et al., 1999). Activity of LTCC can be increased by PKA phosphorylation (Leach et al., 1996; Bünemann et al., 1999). Ca^2+^-dependent inactivation of LTCC can be lessened by CaMKII-phosphorylation, a process activated under oxidizing conditions (Xie et al., 2009). In addition, evidence suggests that the Ca^2+^ channel can be directly activated during oxidative stress, and Cysteine 543 of α1c subunit confers redox sensitivity (Muralidharan et al., 2017; Wilson et al., 2018). Clusters of ≤10 channels are primarily localized in T-tubules in the sites of contact with junctional SR, i.e., dyads, opposing clusters of RyR2 Ca^2+^ release channels (Inoue and Bridge, 2003). Such distribution ensures efficiency of Ca^2+^ release initiation during EC coupling.

### L-Type Ca^2+^ Channel and Cardiac Arrhythmia

Abnormal LTCC function has been implicated in arrhythmogenesis. Gain of function mutations of Cav1.2α1c, as well as loss of function mutation of CaM (reduced Ca^2+^ sensitivity) were linked to hereditary Long QT syndrome type 8 and 14 (Venetucci et al., 2012; Crotti et al., 2013; Marsman et al., 2014). Changes in activation and inactivation parameters leading to widening of so called “window” current were linked to enhanced propensity of reactivation during late phases of AP and thereby generation of early after depolarizations (EADs) (Weiss et al., 2010). Reduction in LTCC expression levels is thought to promote arrhythmogenic Ca^2+^ alternans via reduced fidelity of channel coupling with RyR2s (Harvey and Hell, 2013). Interestingly, reduced LTCC expression levels in disease states are not always reflected by reduced current. For example, in ventricular cardiomyocytes from human failing hearts I_Ca_ was similar to controls, despite of a significant decrease in α1c expression levels, likely due to enhanced phosphorylation by PKA (Chen et al., 2002). Also, fidelity of LTCC-RyR2 coupling can be reduced due to structural remodeling and loss of T-tubules as in hypertrophy, myocardial infarct and HF (Wei et al., 2010).

### L-Type Ca^2+^ Channel in Diabetes

The majority of studies using various models of diabetes did not find statistically significant changes in I_Ca_ with a few exceptions (Pereira et al., 2006; Lu et al., 2007). Pereira et al. (2006) showed that in *db/db* mice (Type 2), the decrease in I_Ca_ originates from a reduced number of channels in the sarcolemma. Similar results were obtained in the Akita mouse model (Type 1, Lu et al., 2007). In both models, steady state activation of I_Ca_ was shifted to more positive voltages which is expected to reduce ‘window current.’ However, in the latter model steady state inactivation was found shifted even further to the right, resulting in larger “window current.” The information as to whether LTCCs in diabetes undergo posttranslational modifications, or if their distribution with regard to RyR2s is altered, is scarce. While Shao et al. (2012) saw no T-tubular remodeling in STZ-diabetic rats, diminished T-tubular density was observed in *db/db* mice (Stølen et al., 2009). These findings, along with changes in LTCC in HF, are summarized in Table 1.

**Table 1:** Changes in LTCC in HF, inherited syndromes and diabetes.

Change in function	Comments	Reference

L-type Ca^2+^ channel (LTCC)		
*Heart failure*		
↔	No change in I_Ca_ No change in basal I_Ca_ but significant increase in phosphorylation of LTCC to maintain it	Mewes and Ravens, 1994; Bodi et al., 2005 Chen et al., 2002
↑	Changes in window current that drive EADs, less Ca^2+^-dependent inactivation of I_Ca_	Bers, 2006; Weiss et al., 2010
↓	Impaired trafficking/reduced abundance in T tubules LTCC-RyR2 coupling fidelity reduced	Hong et al., 2012 Harvey and Hell, 2013
*Inherited syndromes*		
↑	Gain of function mutations in Cav1.2 (Long QT syndrome 8) Loss of function CaM mutation reduced Ca^2+^ sensitivity (Long QT syndrome 14)	Boczek et al., 2013; Ye et al., 2018 Crotti et al., 2013; Marsman et al., 2014
*Type 1 diabetes*		
↔	No change in I_Ca_ in STZ-induced diabetic rats	Smail et al., 2016
↓	Reduced I_Ca_ in Akita(ins2) mice	Lu et al., 2007
*Type 2 diabetes*		
↔	No change in I_Ca_ in Goto-Kakizaki rats	Salem et al., 2013
↓	Reduced number of LTCC channels in sarcolemma in *db/db* mice Reduced I_Ca_ in *db/db* mice	Pereira et al., 2006 Lu et al., 2011

## The Ryanodine Receptor

The cardiac SR Ca^2+^ release channel, RyR2, is a large 2.2 MDa homotetramer consisting of four 565 kDa subunits (Tunwell et al., 1996). While a variety of physiological ligands can modulate RyR2 channel activity including Mg^2+^ and ATP, Ca^2+^ is the primary effector of function (Bers, 2002; Fill and Copello, 2002). During EC coupling, the LTCC-mediated influx of cytosolic Ca^2+^ and increase in [Ca^2+^] drives activation of other RyR2 channels within the cardiomyocyte via CICR (Fabiato, 1985). Activation of single RyR2 clusters consisting of 8–100 channels (Baddeley et al., 2009) generates a local increase in the concentration of cytosolic Ca^2+^, known as a Ca^2+^ spark (Cheng et al., 1996). The summation of Ca^2+^ sparks produced by activated RyR2 clusters throughout the cardiomyocyte leads to a global Ca^2+^ transient that initiates muscle contraction (Cheng et al., 1996).

The positive feedback nature of CICR means it is a self-regenerating process that would be inherently unstable without some mechanism for termination of RyR2-mediated Ca^2+^ release (Fill and Copello, 2002; Kunitomo and Terentyev, 2011). Several candidate mechanisms have been proposed. While it was originally thought that the binding of Ca^2+^ to cytosolic low affinity sites on the channel during Ca^2+^ release inactivated RyR2 channels (Fabiato, 1985), it is now apparent that cytosolic Ca^2+^ plays a limited role in the termination of CICR. There is an accumulation of evidence that demonstrates RyR2 responds to luminal Ca^2+^ concentrations (Sitsapesan and Williams, 1994; Lukyanenko et al., 1996; Györke and Györke, 1998; Terentyev et al., 2002), with Ca^2+^ spark termination occurring when SR [Ca^2+^] falls to a certain level (Brochet et al., 2005; Terentyev et al., 2008; Zima et al., 2008). Depletion may cause unbinding of Ca^2+^ from luminal activation sites and drive closing of the channel [i.e., deactivation (Jiang et al., 2007; Terentyev et al., 2008)]. More recently, in a hypothesis termed ‘induction decay’ or ‘pernicious attrition’ (Gillespie and Fill, 2013; Laver et al., 2013), it was hypothesized that by decreasing local concentrations of cytosolic Ca^2+^, a reduction in unitary current via RyR2 breaks the positive-feedback loop of CICR within a cluster. Another phenomenon related to termination is refractoriness of SR Ca^2+^ release, a period after RyR2 activation and deactivation during which another Ca^2+^ release event cannot occur (Sham et al., 1998; Szentesi et al., 2004; Györke and Terentyev, 2008). While it was thought that the refractory state persists until SR [Ca^2+^] is recovered to a critical level, SR Ca^2+^ load was shown to recover to pre-release levels long before spontaneous Ca^2+^ wave initiation (Belevych et al., 2012). Mechanisms that determine the refractoriness of CICR remain incompletely understood.

While RyR2 channels open and release Ca^2+^ from the SR in response to LTCC-mediated Ca^2+^ influx, channels are not completely closed and have a finite open probability, leading to substantial Ca^2+^ leak during diastole (Bers, 2014; Eisner et al., 2017). This Ca^2+^ leak, measurable as Ca^2+^ sparks, plays an important physiological role in determining the appropriate SR Ca^2+^ load of cardiomyocytes and threshold for SR Ca^2+^ release (Cheng et al., 1996; Eisner et al., 2017).

Multiple accessory proteins have been shown to coimmunoprecipitate with RyR2, indicative that the channels exist as large macromolecular complexes (Bers, 2004; Meissner, 2017). The Ca^2+^ binding protein calmodulin (CaM) directly associates with and regulates RyR2 channels (Samsó and Wagenknecht, 2002; Bers, 2004), while auxilliary proteins CSQ, triadin (TRDN) and junctin (JUN) form the luminal Ca^2+^ sensor of RyR2 within the SR (Györke et al., 2004; Györke and Terentyev, 2008). FK506 binding protein 12.6 (FKBP12.6) is also an accessory protein of RyR2, and the interaction was proposed to stabilize the channel, preventing spontaneous Ca^2+^ release and SR Ca^2+^ leak, although this phenomenon was not observed by many others (Marx et al., 2000; Prestle et al., 2001; George et al., 2003; Goonasekera et al., 2005; Wehrens et al., 2005).

During β-adrenergic stimulation as part of the ‘fight or flight’ response, several EC coupling proteins are targets for posttranslational modification, with phosphorylation well established as a regulatory mechanism modulates ion channel activity with positive chronotropic and inotropic effects to enhance cardiac function (Bers, 2002). The RyR2 channel complex includes a network of associated kinases (protein kinase A (PKA) and Ca^2+^-calmodulin dependent protein kinase II (CaMKII)) and phosphatases (PP1, PP2A, and PP2B) that dynamically and reversibly modulate its phosphorylation state (Belevych et al., 2011a; Niggli et al., 2013; Terentyev and Hamilton, 2016). Three major phosphorylation sites have been identified – PKA-specific Serine 2808 (S2808) and Serine 2031 (S2031), and CaMKII-specific Serine 2814 (S2814) (Witcher et al., 1991; Wehrens et al., 2004b; Xiao et al., 2005).

Another major posttranslational modification of RyR2 function is oxidation (Zima and Mazurek, 2016). To maintain a fine-tuned balance of reduced and oxidized proteins within the cardiomyocytes, there are multiple sources of ROS as well as antioxidant defense components (Xu et al., 1998). There are approximately 21 cysteine residues within RyR2 that are reduced under physiological conditions, and the channel is known to be susceptible to reversible redox modification (Xu et al., 1998; Dulhunty et al., 2000). At the single channel level, oxidation of RyR2 increases channel open probability and increases the sensitivity to activating Ca^2+^, while reducing agents have opposite effects (Boraso and Williams, 1994; Xu et al., 1998; Salama et al., 2000).

### The Ryanodine Receptor and Cardiac Arrhythmia

Under pathophysiological conditions, diastolic Ca^2+^ leak from the SR is increased, thus exceeding the critical threshold level and increasing RyR2 channel activation, which in turn activates other channels and results in proarrhythmic diastolic Ca^2+^ waves (Cheng et al., 1996). This subsequently activates NCX1, driving a net inward current that gives rise to delayed after depolarizations (DADs) and arrhythmias (Ferrier et al., 1973; Mechmann and Pott, 1986; Landstrom et al., 2017). Evidence suggests that increased refractory period shortening, thus increased RyR2-mediated SR Ca^2+^ leak, promotes Ca^2+^-dependent arrhythmias in failing hearts (Belevych et al., 2012; Brunello et al., 2013; Cooper et al., 2013).

Abnormal Ca^2+^ release is the driving force for arrhythmia observed in patients with catecholaminergic polymorphic ventricular tachycardia (CPVT), a condition characterized by pathogenic mutations in RyR2 (Priori et al., 2002; Priori and Chen, 2011), as well as in associated accessory proteins including CSQ (Terentyev et al., 2006; Nyegaard et al., 2012; Roux-Buisson et al., 2012). The condition presents under conditions of enhanced catecholaminergic drive, in the absence of any structural defects in the heart (Priori et al., 2002; Tester et al., 2005). Premature ventricular contractions (PVCs) that often manifest during exercise or periods of stress can degenerate into polymorphic or bidirectional ventricular tachycardia (VT) or fibrillation (VF) and subsequently lead to SCD. Mutations in RyR2 associated with CPVT usually cause increased SR Ca^2+^ leak that is exacerbated by β-adrenergic stimulation, but both gain- and loss-of-function mutations have been reported (Wehrens et al., 2003; Loaiza et al., 2013; Zhao et al., 2015; Landstrom et al., 2017; Uehara et al., 2017). Mutations in CSQ cause reduction or complete loss of protein expression, and without CSQ-mediated Ca^2+^ buffering within the SR, RyR2 channels are prone to spontaneous activation, offering a trigger for arrhythmia (Yano and Zarain-Herzberg, 1994; Lahat et al., 2001; Knollmann et al., 2006; Faggioni et al., 2012).

The significance of altered PKA-mediated phosphorylation of RyR2 function in the pathogenesis of cardiac arrhythmia remains controversial. Hyperphosphorylation of RyR2 at S2808 was originally hypothesized to cause dissociation of FKBP12.6, destabilizing the channel and thus increasing open channel probability (Marx et al., 2000). However, the significance of both phosphorylation at this site and the role of PKA-mediated phosphorylation on the function of RyR2 remains a disputed subject (Jiang et al., 2002; Benkusky et al., 2007; Curran et al., 2007; Belevych et al., 2011b; Bovo et al., 2017). The role of CaMKII-mediated Ry2 phosphorylation in modulating channel function is more strongly supported, with consensus that activation of CaMKII as opposed to PKA increases SR Ca^2+^ leak (Ai et al., 2005; Curran et al., 2007; Niggli et al., 2013), although this is not universal (Wehrens et al., 2006; Yang et al., 2007). It is also well established that chronic CaMKII activity in cardiac disease is a major regulator of RyR2 function with arrhythmogenic consequences (Ai et al., 2005; Zhang et al., 2005; Terentyev et al., 2009; Belevych et al., 2012; Respress et al., 2012; Uchinoumi et al., 2016). Additionally, oxidation as a posttranslational modification may alter RyR2 function in cardiac disease. In the healthy heart, oxidation may serve to transiently enhance Ca^2+^ release, increasing cardiac output (Niggli et al., 2013). However, in conditions of severe oxidative stress such as HF, increased RyR2 oxidation by ROS can lead to RyR2 activation and increased proarrhythmic SR Ca^2+^ leak (Mochizuki et al., 2007; Belevych et al., 2011a,b). Modulation of RyR2 activity by ROS can also be indirect, via the oxidation of CaMKII and subsequent increased CaMKII-mediated phosphorylation of the channel (Chelu et al., 2009; Anderson, 2015).

The SR Ca^2+^ load is thought to be a critical factor of cardiac alternans, a repetitive beat-to-beat fluctuation in cellular repolarization at a constant heart rate that closely linked to development of ventricular arrhythmias (Edwards and Blatter, 2014). Any impairment in the refractoriness of RyR2-mediated Ca^2+^ release or the recovery after channel inactivation may facilitate the onset of alternans, due to a reduction in the subsequent Ca^2+^ transient amplitude (Edwards and Blatter, 2014). These properties of RyR2 have been shown to be a major component underlying generation of alternans in both computational and experimental studies (Sobie et al., 2006; Nivala and Qu, 2012; Shkryl et al., 2012; Alvarez-Lacalle et al., 2013; Sun et al., 2018).

### The Ryanodine Receptor in Diabetes

It is well established that increased SR Ca^2+^ leak due to enhanced RyR2 channel activity significantly contributes to arrhythmogenic potential in diabetic cardiomyopathy.

Injection of STZ destroys insulin-producing β cells and has long been used for the generation of Type 1 diabetes phenotypes. Early studies of RyR2-mediated Ca^2+^ release in diabetes utilized STZ-diabetic rat models. Work of Yu et al. (1994) showed that cardiomyocytes from STZ-diabetic rats had reduced maximum rates of shortening and relengthening, as well as depressed SR Ca^2+^ content. Using [^3^H] ryanodine binding assay as an indication of RyR2 channel functionality, isolated SR membranes from diabetic rats showed reduced high-affinity binding sites. Bidasee et al. (2001) also reported decreased [^3^H] ryanodine binding in 6-week post STZ injection, but posited this was due to dysfunctional RyR2 rather than a decrease in protein expression or mRNA level as observed in Teshima et al. (2000) and Choi et al. (2002). Interestingly, Zhao et al. (2014) found that Ca^2+^ spark frequency showed a gradual decline in correlation with progression of STZ-diabetes, with significant differences between 4-week and 12-week post-injection groups.

Yaras et al. (2005) importantly showed increased Ca^2+^ spark frequency in cardiomyocytes from STZ-diabetic rat, with a reduced Ca^2+^ transient amplitude and depressed SR Ca^2+^ loading. This was accompanied by significantly increased phosphorylation of RyR2 at S2808 and a ∼40% decrease in FKBP12.6 association. Similar findings were reported by other groups, where these phenomena were suggested to underscore depressed SR Ca^2+^ release in STZ-diabetic cardiomyocytes (Shao et al., 2007, 2009; Tuncay et al., 2014), indicative that Ca^2+^ leak via hyperactive RyR2 contributes to the disease phenotype. Later work showed that exercise training for 4 weeks could attenuate this, reducing S2808 phosphorylation and increase levels of FKBP12.6 expression (Shao et al., 2009). However, the functional role of PKA-mediated RyR2 phosphorylation remains controversial and many studies have shown phosphorylation at the S2808 site does not modulate channel activity in other cardiac disease states (Xiao et al., 2004; Guo et al., 2010). An increase in endogenous CaMKII-mediated phosphorylation of RyR2 has also been implicated in the aberrant Ca^2+^ handling observed in STZ-diabetic rats (Netticadan et al., 2001) and *db/db* mice (Stølen et al., 2009). Conversely, Tian et al. (2011) posited that gain-of-function changes in RyR2 function observed in single channel recordings were independent of phosphorylation at either S2808 or S2814 sites. Instead, the increase in open channel probability and 20% reduction in conductance was attributed to increased responsiveness to cytoplasmic activators including Ca^2+^, alterations in the threshold for activation by luminal Ca^2+^, and a blunted response to physiological inhibitors.

Other posttranslational modifications of RyR2 in Type 1 diabetes have also been suggested to underscore channel dysfunction. Bidasee et al. (2003a) suggested RyR2 dysfunction, evidenced by decreased [^3^H] ryanodine binding in STZ-diabetic rats, was in part due to formation of disulfide bonds between adjacent sulfhydryl groups. The same group also showed that non-cross-linking advanced glycation end products (AGEs) on RyR2 are significantly increased in diabetic heart tissue, and this increase could be partially attenuated with insulin treatment (Bidasee et al., 2003b). Extensive carbonylation of RyR2 by increased reactive carbonyl species (RCS) in STZ- diabetic rats was suggested to reduce the responsiveness of RyR2 to cytoplasmic Ca^2+^. While expression levels of RyR2 remained unchanged, there was an increase in non-functional RyR2 channels, but also an increase in the activity of others. The increased heterogeneity of RyR2 channels was posited to increase spontaneous and dyssynchronous SR Ca^2+^ release in isolated cardiomyocytes, thus providing a trigger for arrhythmia. Treatment of diabetic rats with RCS scavengers attenuated spontaneous SR Ca^2+^ release, reduced RyR2 carbonylation and normalized channel functionality.

Fewer studies have investigated changes in RyR2-mediated Ca^2+^ handling in models of Type 2 diabetes. In the non-failing myocardium of type 2 diabetic patients RyR2 protein expression was decreased, while mRNA levels were decreased in the Goto-Kakizaki model (Reuter et al., 2008; Gaber et al., 2014). In a prediabetic model of metabolic syndrome, whereby dogs were chronically fed a high fat diet (HFD), phosphorylation of RyR2 at S2808 was significantly elevated and the channel’s ability to bind [^3^H] ryanodine significantly depressed in the ventricles compared to healthy controls, while no changes in RyR2 mRNA or protein expression were observed (Dinçer et al., 2006). Okatan et al. (2016) also observed increased RyR2 phosphorylation at S2808 in rats with high sucrose diet-induced metabolic syndrome, accompanied by reduced FKBP12.6 expression. Through studies of electric-field stimulated intracellular Ca^2+^ handling, cardiomyocytes isolated from rats with metabolic syndrome showed significantly increased SR Ca^2+^ leak, depressed SR Ca^2+^ loading and reduced Ca^2+^ transient amplitude vs. controls. This data is suggestive that alterations in RyR2 function and SR Ca^2+^ release may be an important mechanism of early cardiac dysfunction in insulin resistance and diabetes development.

Abnormal intracellular lipid concentration is a hallmark of both obesity and diabetes, and Joseph et al. (2016) recently studied Ca^2+^ handling in cardiomyocytes from a transgenic model of cardiac lipid overload, with peroxisome proliferator-activated receptor-γ (PPARg) overexpression. This revealed increased Ca^2+^ spark activity compared to controls that could be reduced by application of antioxidant mitoTEMPO. A significant increase in mitochondrial oxidative stress was suggested to increase RyR2 oxidation and subsequent SR Ca^2+^ release. In 8-week mice with HFD-induced obesity, Sánchez et al. (2018) reported a shift in the distribution of single RyR2 channel responsiveness to activating cytosolic [Ca^2+^], whereby channels were much more active to those isolated from control mice. No changes were observed in RyR2 expression levels or phosphorylation status at S2808 or S2814 sites. Instead, this phenomenon was attributed to significantly increased RyR2 oxidation in HFD-mice, implicating the diabetes-related increase in oxidative stress in abnormal Ca^2+^ handling. Changes in RyR2 in HF and diabetes are summarized in Table 2.

**Table 2:** Changes in RyR2 in HF, inherited syndromes and diabetes.

Change in function	Comments	Reference

**Ryanodine Receptor (RyR2)**		
*Heart failure*		
↑	Increases diastolic SR Ca^2+^ leak, resulting in diastolic Ca^2+^ waves Increased phosphorylation (S2808) Decreased FKBP12.6 association Increased phosphorylation (S2031) Increased phosphorylation (S2814) Increased oxidation Other posttranslational modifications	Belevych et al., 2011b; Cheng et al., 1996 Marx et al., 2000 Marx et al., 2000; Wehrens et al., 2003; Wehrens et al., 2005 Xiao et al., 2005 Ai et al., 2005; Curran et al., 2007; Terentyev et al., 2009; Sossalla et al., 2010; Belevych et al., 2011b; Respress et al., 2012; Dries et al., 2018 Mochizuki et al., 2007; Terentyev et al., 2008; Belevych et al., 2011b Xu et al., 1998; Barouch et al., 2002
*Inherited syndromes*		
↑	CPVT; mostly gain of function RyR2 mutations, or mutations in accessory proteins	Yano and Zarain-Herzberg, 1994; Lahat et al., 2001; Priori et al., 2002; Terentyev et al., 2006; Roux-Buisson et al., 2012
*Type 1 diabetes*		
↑	Decreased protein expression/mRNA level in STZ-diabetic rats (increased activity due to posttranslational modification) Increased PKA-mediated phosphorylation (S2808) in STZ-diabetic rats Decreased FKBP12.6 association in STZ-diabetic rats Increased CaMKII-mediated phosphorylation (S2814) in STZ-diabetic rats Other posttranslational modification in STZ-diabetic rats (oxidation, carbonylation, AGEs) Change in sensitivity to cytosolic or luminal Ca^2+^ activation in STZ diabetic rats	Teshima et al., 2000; Yaras et al., 2005; Zhao et al., 2014; Chou et al., 2017 Netticadan et al., 2001; Yaras et al., 2005; Shao et al., 2007, 2009 Yaras et al., 2005; Shao et al., 2007, 2009; Tuncay et al., 2014; Zhao et al., 2014 Netticadan et al., 2001; Shao et al., 2009 Bidasee et al., 2003a,b; Shao et al., 2012 Tian et al., 2011; Shao et al., 2012
*Type 2 diabetes*		
↑	Reduced protein expression in nonfailing diabetic human myocardium, with increased phosphorylation Reduced mRNA levels in Goto-Kakizaki rats Reduced protein expression levels in PPARg mice with lipid overload Increased PKA-mediated phosphorylation (S2808) in high-sucrose diet rats and HFD dogs Increased oxidation in PPARg mice with lipid overload and HFD mice Change in sensitivity to cytosolic or luminal Ca^2+^ activation in HFD mice	Reuter et al., 2008 Gaber et al., 2014 Joseph et al., 2016 Dinçer et al., 2006; Okatan et al., 2016 Joseph et al., 2016; Sánchez et al., 2018 Sánchez et al., 2018

## Sarco/Endoplasmic Reticulum Ca^2+^-Atpase

For relaxation to occur, intracellular [Ca^2+^] is decreased primarily via sequestration into the SR by SERCa2a, the primary cardiac SERCa isoform (Bers, 2002). While SERCa2a interacts with multiple proteins (including calreticulin, HRC, PP1, S100A, sarcolipin, SUMO), the most important regulator of function is phospholamban (PLB) (Kranias and Hajjar, 2012). Unphosphorylated PLB has an inhibitory effect on SERCa2a activity, lowering affinity of the pump for Ca^2+^. Conversely phosphorylation of PLB, either by PKA at Serine 16 (S16) or CaMKII at Threonine 17 (T17), relieves SERCa2a inhibition and increases activity (Simmerman et al., 1986). Oxidative thiol modification of SERCa2a at Cysteine 674 also enhances function (Lancel et al., 2009). Upregulated SERCa2a function is the primary mechanism for positive lusitropic (accelerated relaxation) and inotropic (increased contraction) responses during β-adrenergic stimulation (Fearnley et al., 2011; Vervliet et al., 2018).

### Sarco/Endoplasmic Reticulum Ca^2+^-ATPase and Cardiac Arrhythmia

In HF, impaired SERCa2a expression and activity blunts Ca^2+^ transient amplitude and rate of decay (Winslow et al., 1999). Reduced sequestration of Ca^2+^ into the SR drives Ca^2+^ extrusion from the cardiomyocyte via NCX1. This generates a net inward depolarizing current that can prolong action potential and thus facilitate triggered activity (O’Rourke et al., 1999; Bers et al., 2002). Diminished SR Ca^2+^ uptake by SERCa2a is also associated with the initiation of cardiac alternans (Merchant and Armoundas, 2012; Nivala and Qu, 2012).

While it may seem counterintuitive to increase SR Ca^2+^ uptake as a therapeutic strategy, given that SR Ca^2+^ overload may exacerbate SR Ca^2+^ leak through RyR2, accumulated evidence suggests the contrary. Upregulation of SERCa2a in small and large animal studies has been shown to protect against development of arrhythmias, improve contractile function and normalize intracellular Ca^2+^ handling (Meyer and Dillmann, 1998; del Monte et al., 2001, 2004; Suarez et al., 2004; Prunier et al., 2008; Lyon et al., 2011; Fernandez-Tenorio and Niggli, 2018). Multicenter SERCa2a gene therapy trials in humans with HF have been completed, but with limited success (Jaski et al., 2009; Jessup et al., 2011; Zsebo et al., 2014; Greenberg et al., 2016). Increased SERCa2a activity also suppressed cardiac alternans in both computational models and experimental studies (Cutler et al., 2009; Stary et al., 2016).

### Sarco/Endoplasmic Reticulum Ca^2+^-ATPase in Diabetes

Diminished SR Ca^2+^ uptake has been identified as a primary mechanism for decreased cardiac contractility observed in diabetic cardiomyopathy (Ganguly et al., 1983). Dysfunction of SERCa2a has been established at early stages of type 1 diabetes development and is primarily ascribed to decreased mRNA levels or expression of the protein, resulting in reduced Ca^2+^ transient amplitudes and a slower rate of transient decay (Ganguly et al., 1983; Trost et al., 2002; Bidasee et al., 2004; Hu et al., 2005; Lacombe et al., 2007). Increased oxidative stress and intracellular ROS concentrations in diabetic hearts can reduce SERCa2a activity by oxidizing Cysteine 674, as well as interfering with the ATP binding site (Xu et al., 1997; Ying et al., 2008). Other molecular mechanisms suggested to drive SERCa2a downregulation include increased carbonylation, glycation and O-GlcNAcylation (Bidasee et al., 2004; Hu et al., 2005; Shao et al., 2011). Changes in PLB expression and phosphorylation have also been reported in STZ-diabetic rats, but this finding is not universal (Wold et al., 2005). Gene therapy with recombinant PLB antibody, which could mimic PLB phosphorylation thus activate SERCa2a, was also shown to increase the rate of whole heart relaxation, contraction and pressure development in a diabetic mouse model and cardiomyopathic hamsters (Meyer et al., 2004; Dieterle et al., 2005).

The pathophysiological role of SERCa2a in cardiomyopathy observed in type 2 diabetes remains to be fully elucidated, although activity is mostly reduced in various models (Zarain-Herzberg et al., 2014). Decreased SERCa2a mRNA levels were observed in the obese *fa/fa* rats while levels of protein, but not mRNA, were reduced in Otsuka Long-Evans Tokushima Fatty (OLETF) rats. No changes in protein expression were observed in the *db/db* mouse, but rather an increased PLB expression was posited to account for diminished SR Ca^2+^ uptake in this model. Stølen et al. (2009) reported reduced SERCa2a protein expression, increased PLB phosphorylation and overall reduced SERCa2a Ca^2+^ uptake in *db/db* mice. Conversely, increased expression of SERCa2a and reduced PLB mRNA was observed in ZDF rats, which could be further increased with insulin treatment in a concentration-dependent manner (Fredersdorf et al., 2012). Upregulation of SR Ca^2+^ uptake via SERCa2a may offer protection in early phases of disease development, countering volume overload and impaired relaxation (Fredersdorf et al., 2012; Zarain-Herzberg et al., 2014). Changes in SERCa2a expression and function in both HF and diabetes are summarized in Table 3.

**Table 3:** Changes in SERCa2a in HF, inherited syndromes and diabetes.

Change in function	Comments	Reference

**Sarco/endoplasmic reticulum Ca^2+^ ATPase (SERCa2a)**		
*Heart failure*		
↓	Depressed activity Reduced mRNA levels/protein expression Decreased PLB expression Decreased PLB phosphorylation Increased PLB phosphorylation Dissociation of SUMO1	Pieske et al., 1995; Schmidt et al., 1998, 1999; Currie and Smith, 1999; Jiang et al., 2002 Kiss et al., 1995; Currie and Smith, 1999; Armoundas et al., 2007 O’Rourke et al., 1999; Jiang et al., 2002; Armoundas et al., 2007 Schwinger et al., 1998, 1999 Currie and Smith, 1999 Schmidt et al., 1999
*Inherited syndromes*		
↓	PLB mutation, reduced SERCa2a activity	Schmitt et al., 2003
↑	PLB deletion mutation, enhanced SERCa2a activity	Haghighi et al., 2003, 2006
*Type 1 diabetes*		
↓	Reduced SERCa2a activity in alloxan and STZ-induced diabetic rats Decreased mRNA levels/protein expression levels in STZ-diabetic rats Decreased SERCa2a expression, increased CaMKII phosphorylation of SERCa2a and PLB in STZ-diabetic rats Increased oxidation in diabetic pig aorta Increased carbonylation, glycation and O-GlcNAcylation in STZ-diabetic rats	Lopaschuk et al., 1983; Allo et al., 1991 Teshima et al., 2000; Choi et al., 2002; Trost et al., 2002; Bidasee et al., 2004; Hu et al., 2005; Lacombe et al., 2007 Netticadan et al., 2001 Ying et al., 2008 Bidasee et al., 2004; Hu et al., 2005; Shao et al., 2011
*Type 2 diabetes*		
↑	Increased SERCa2a expression but reduced PLB mRNA in Zucker rats	Fredersdorf et al., 2012
↓	Slowed SR Ca^2+^ uptake without changes in SERCa2a expression but increased PLB phosphorylation in rats fed high starch/sucrose Decreased mRNA levels/protein expression levels in obese *fa/fa* and OLETF rats No change in SERCa2a expression but increased PLB expression in *db/db* mouse Reduced SERCa2a expression but increased in phosphorylation of PLB in PPARg mice Reduced SERCa2a expression but enhanced CaMKII-mediated phosphorylation of PLB in *ob/ob* mice.	Wold et al., 2005 Zarain-Herzberg et al., 2014 Zarain-Herzberg et al., 2014 Joseph et al., 2016 Stølen et al., 2009

## Na^+^/Ca^2+^ Exchanger

To maintain the cardiac contraction cycle, equal amount of Ca^2+^ that enters the cell though LTCCs must be removed to the extracellular milieu. The Na^+^/Ca^2+^ exchanger is the main route for Ca^2+^ extrusion from myocytes. Cardiac NCX (NCX1, 110 kDa) is activated by intracellular Ca^2+^ in submicromolar range transporting one Ca^2+^ ion in exchange to thee Na^2+^ ions (Despa and Bers, 2013). In the early phases of action potential at voltages more positive than reversal potential of NCX1, a small amount of Ca^2+^ enters the cell. This “primes” RyR2 clusters for activation during subsequent openings of LTCCs, enhancing efficiency of CICR (Neco et al., 2010). At later stages of action potential during the Ca^2+^ transient, NCX1 works in a forward mode extruding Ca^2+^, and, since it is electrogenic, contributes to depolarization. Increase in intracellular [Na^2+^] can significantly increase NCX1-mediated Ca^2+^ influx and reduce removal. Although NCX1 activity was demonstrated being increased in the presence of oxidants (Kuster et al., 2010), evidence also exists that enhanced production of ROS leads to NCX1 inhibition (Liu and O’Rourke, 2013).

### Na^+^/Ca^2+^ Exchanger and Cardiac Arrhythmia

Enhanced expression and activity of NCX1 is thought to be one of the major causes of increased arrhythmogenesis in HF (Pogwizd et al., 1999; Bers et al., 2002). NCX1 translates intracellular [Ca^2+^] during spontaneous Ca^2+^ waves into depolarizations, i.e., DADs, which can lead to activation of Na^2+^ channels and extrasystolic action potentials. During systole enhanced NCX1 prolongs APD allowing LTCCs to reactivate thereby contributing to generation of EADs. Increased NCX1-mediated Ca^2+^ influx during reverse mode due to increased intracellular [Na^2+^] characteristic of HF or ischemia may increase cytosolic Ca^2+^ and thereby activity of RyR2s (Satoh et al., 2000; Szepesi et al., 2015). Pharmacological inhibition of NCX1 substantially reduced triggered activity in various models of ventricular arrhythmia with perturbed Ca^2+^ homeostasis (Bourgonje et al., 2013; Jost et al., 2013; Nagy et al., 2014; Zhong et al., 2018). Experiments using mouse ventricular myocytes with reduced NCX1 demonstrated that genetic inhibition of NCX1 suppressed arrhythmogenic after depolarizations (Bögeholz et al., 2015), while transgenic overexpression promoted generation of EADs due to prolonged repolarization and spontaneous action potentials (Pott et al., 2012).

### Na^+^/Ca^2+^ Exchanger in Diabetes

The levels and activity of NCX1 vary in different diabetes models. Alloxan or STZ- injected mice with diabetes type 1 showed reduction in NCX1 activity, reduced expression and mRNA levels (Makino et al., 1987; Pierce et al., 1990; Golfman et al., 1998; Hattori et al., 2000). A number of studies shows increased intracellular [Na^+^] in myocytes from diabetic hearts which enhances NCX1-mediated Ca^2+^ influx and reduces extrusion (Doliba et al., 2000; Wickley et al., 2007), but reduced NCX1 expression levels have also been reported (Bilginoglu et al., 2013). Belke et al. (2004) using the diabetes type 2 *db/db* mouse model showed no difference in NCX1 activity. In *db/db* mice, Stølen et al. (2009) reported increased NCX1 function. Insulin-resistant sucrose-fed rats with diastolic dysfunction showed normal expression and function of NCX1 (Wold et al., 2005). No changes in I_NCX_ were reported in high fat diet mouse model (Ricci et al., 2006). An increase in mRNA levels of NCX1 was shown in human patients with diabetes type 2 (Ashrafi et al., 2017). A similar increase was demonstrated in the mouse model with lipid overload (Joseph et al., 2016) and diabetes type 1 Akita(ins2) mouse model of cardiomyopathy (LaRocca et al., 2012). Alterations in NCX1 expression and function in HF and diabetes are summarized in Table 4.

**Table 4:** Changes in NCX1 in HF, inherited syndromes and diabetes.

Change in function	Comments	Reference

**Na^+^/Ca^2+^-exchanger (NCX1)**		
*Heart failure*		
↔	No change in NCX1 function in end-stage human HF	Piacentino et al., 2003
↑	Increased protein expression and enhanced activity Enhanced reverse mode activity, with Ca^2+^ entry into the myocyte	Pogwizd et al., 1999, 2001; O’Rourke et al., 1999; Bers et al., 2002; Ottolia et al., 2013 Despa et al., 2002; Weber et al., 2003
*Inherited syndromes*		
	N/A	
*Type 1 diabetes*		
↑	Increased activity Increased mRNA levels in Akita(ins2) mice	Doliba et al., 2000; Wickley et al., 2007 LaRocca et al., 2012
↓	Depressed activity in STZ-diabetic rats Reduced mRNA levels/protein expression in STZ-diabetic rats	Pierce et al., 1990; Allo et al., 1991; Hattori et al., 2000 Hattori et al., 2000
*Type 2 diabetes*		
↔	No change in NCX1 activity/protein expression in *db/db* mice, high-sucrose diet rats, HFD mice	Belke et al., 2004; Wold et al., 2005; Ricci et al., 2006
↑	Increased mRNA levels in human left ventricle Increased mRNA levels in PPAR mice with lipid overload Increased activity in *db/db* mice	Ashrafi et al., 2017 Joseph et al., 2016 Stølen et al., 2009

## Mechanisms of Ca^2+^ Dependent Arrhythmia in Diabetes

The enhanced propensity to ventricular tachyarrhythmias in diabetic and obese patients is well established (Tse et al., 2016). The arrhythmic potential increases relatively early in the course of development of diabetic cardiomyopathy before the onset of systolic dysfunction. Lacombe et al. (2007) showed dramatic increase in EADs and DADs in ventricular myocytes from diabetic rats with diastolic dysfunction 8 weeks after STZ injection. Sommese et al. (2016) demonstrated enhanced arrhythmogenesis *in vivo* in fructose rich diet (FRD) prediabetic mice. The authors showed increased activity of RyR2 manifested in enhanced frequency of spontaneous Ca^2+^ waves in field-stimulated myocytes. Mice expressing SR-targeted CaMKII inhibitor peptide AIP showed less ectopic activity that WT FRD mice, and in rats FRD-mediated increase in pro-arrhythmic spontaneous Ca^2+^ release was eliminated by incubation of myocytes with pharmacological inhibitor of CaMKII KN93 or antioxidant Tempol. The authors conclude that RyR2 phosphorylation by ROS-activated CaMKII at CaMKII site S2814 plays a major role in diabetes-related arrhythmogenesis. Interestingly, the authors did not find changes in RyR2 phosphorylation at PKA site S2808. Earlier work by Erickson et al. (2013) linked diabetic hyperglycemia-mediated activation of CaMKII with Ca^2+^-dependent triggered activity in rats. The authors proposed that acute hyperglycemia causes covalent modification of CaMKII by O-linked *N*-acetylglucosamine (O-GlcNAc), resulting in prolonged activation of CaMKII and subsequent increase in RyR2 CaMKII phosphorylation and thereby its activity.

Diabetes is associated with oxidative stress and mitochondrial dysfunction plays significant role in enhanced production of ROS (Akar, 2013; Xie et al., 2013; Teshima et al., 2014). It is postulated that a micro-domain between the SR and mitochondria allows for control by Ca^2+^ of mitochondrial function and SR Ca^2+^ handling machinery by mitochondrial ROS (Ruiz-Meana et al., 2010; Eisner V. et al., 2013; Lopez-Cristosto et al., 2017). The close proximity of mitochondria to SR Ca^2+^ release sites (∼37–270 nm; Sharma et al., 2000) facilitates not only mitochondrial Ca^2+^ uptake and subsequent alterations in mitochondrial function (Csordás et al., 2001; Dorn and Maack, 2013), but also ROS-mediated modification of RyR2 and SERCa2a, both of which are redox sensitive (Zima and Blatter, 2006). In the PPARg overexpression mouse, it was demonstrated that oxidative stress due to mitochondrial dysfunction causes increased SR Ca^2+^ leak by oxidizing RyR2 channels (Joseph et al., 2016). This promoted ventricular ectopy, which was significantly reduced *in vivo* by a mitochondrial-targeted antioxidant mitoTEMPO. Sánchez et al. (2018) reported increased incidences of PVCs and non-sustained VT in HFD-mice vs. controls and this phenomenon was attributed to significantly increased RyR2 oxidation. When antioxidant apocynin was provided in the drinking water, appearance of ventricular arrhythmias in this model was completely abolished. Interestingly, results from Fauconnier et al. (2007) and Llano-Diez et al. (2016) suggest that in *ob/ob* and HFD mice at certain stages of disease development, mitochondrial ROS emission is reduced. Fauconnier et al. (2007) proposed that during prolonged exposure to increased fatty acid levels due to the switch in mitochondrial substrate utilization, cardiomyocytes from the *ob/ob* mouse adapt to preferential use of fatty acids for metabolism, so further exposure to fatty acids improved intracellular Ca^2+^ homeostasis in this model.

Increased NCX1 activity has also been identified as a mechanism promoting arrhythmia via enhancement of depolarization during spontaneous SR Ca^2+^ release events (Pott et al., 2011). Joseph et al. (2016) showed that in the PPARg overexpression model, NCX1 was upregulated. Importantly, in left ventricular tissue samples from type 2 diabetes human patients NCX1 expression was found significantly higher than in control patients with hypertrophy (Ashrafi et al., 2017).

Direct evidence that diabetes-related aberrant Ca^2+^ homeostasis can contribute to reentrant mechanisms of arrhythmia was obtained by Chou et al. (2017) who showed that optically mapped hearts from obese *db/db* mice exhibit concordant Ca^2+^/Vm alternans at lower stimulation frequencies than controls. The authors attributed it to lower expression levels of RyR2 and enhanced activity of CaMKII. Interestingly, previous studies using this model showed that the expression levels of LTCCs are substantially reduced (Pereira et al., 2006), while RyR2- mediated leak enhanced; and SERCA2a function severely depressed because of enhanced expression of PLB (Belke et al., 2004). Indeed, reduced fidelity of LTCC/RyR2 coupling and impaired ability to re-sequester Ca^2+^ into the SR are thought to be major underlying causes for beat-to-beat alternations of intracellular Ca^2+^ transient amplitude (Edwards and Blatter, 2014).

## Therapeutic Strategies to Improve Ca^2+^ Homeostasis in Diabetes

Profound changes in cardiac Ca^2+^ homeostasis were reported in multiple animal models of diabetes at various states of disease progression, yet mechanisms underlying Ca^2+^ mishandling remain to be fully understood. While many experimental studies have sought to elucidate mechanisms underlying the enhanced propensity for arrhythmia in insulin-dependent models of type 1 diabetes, those driving arrhythmogenesis in type 2 diabetes are far less investigated. Given the prevalence of acquired diabetes is drastically increasing due to higher rates of obesity in the developed world, future studies are necessary to understand this complex phenotype.

### Insulin Replacement

Strategies to manage diabetes include insulin replacement and aerobic exercise regimes (Winnick et al., 2008). Insulin elicits positive inotropic effects on the myocardium, inducing SERCa2a activity (Pierce et al., 1985), while endurance exercise has been shown to improve intracellular Ca^2+^ cycling and protect against oxidative stress in diabetic animal models (Kim et al., 1996; Shao et al., 2009; Stølen et al., 2009). Combined therapy was shown to increase expression of Ca^2+^ handling proteins, restore Ca^2+^ handling and improve basal cardiac function in type 1 diabetic rats (Le Douairon Lahaye et al., 2012; da Silva et al., 2015). However, due to the multifaceted nature of the disease, diabetes remains a prime risk factor for cardiovascular disease despite beneficial effects of these frontline treatments (Leon and Maddox, 2015).

### Inhibition of the Renin-Angiotensin System

During the progression of diabetic cardiomyopathy, hyperglycemia is known to increase activity of the renin-angiotensin system (RAS) (Sechi et al., 1994; Ohishi, 2018). Angiotensin II has been shown to have direct effects on cardiomyocytes, with activation of its receptor (AT1) increasing generation of oxidants and increasing intracellular [Ca^2+^], as well as activating protein kinase C (PKC) and PKA (Malhotra et al., 1997; de Lannoy et al., 1998; Dostal, 2000; Raimondi et al., 2004). Angiotensin-converting enzyme (ACE) inhibitors and AT1 blockers are used to prevent hypertension and cardiovascular disease in diabetic patients (Mancia et al., 2013; Singh et al., 2018). Antagonism of angiotensin II was demonstrated to alleviate diminished SERCa2a activity in HF models (Okuda et al., 2004; Gassanov et al., 2006), while administration of AT1 blockers to STZ-diabetic rat cardiomyocytes reduced cellular oxidative stress as well as phosphorylation levels of RyR2, improving Ca^2+^ homeostasis (Privratsky et al., 2003; Yaras et al., 2007; Ozdemir et al., 2009).

### β-Blockade

Current and conventional treatments of diabetes are limited in terms of preventing ventricular arrhythmias and SCD. Blockade of the β-adrenergic stimulation cascade remains a primary treatment of HF in a bid to reduce arrhythmogenic responses, including those of Ca^2+^ handling proteins (Klapholz, 2009; Rehsia and Dhalla, 2010). However, use of antagonists in diabetic patients remains controversial, given β-blockers have been associated with increased risk for cardiovascular events in diabetic patients and can have hypoglycemic side effects (Casiglia and Tikhonoff, 2017; Tsujimoto et al., 2017).

### Targeting LTCC or NCX1

As it stands, inhibition of LTCC or NCX1 does not appear to be an appropriate strategy in ameliorating Ca^2+^ mishandling in diabetic hearts. Current density of LTCC is reported as unaltered or reduced in diabetic models (Pereira et al., 2006; Lu et al., 2007). A reduction of I_Ca_ in this setting would diminish the trigger for RyR2-mediated Ca^2+^ release and thereby may exacerbate systolic dysfunction and increase propensity to Ca^2+^ alternans. Pharmacological inhibition of NCX1 is generally viewed as beneficial since it reduces Ca^2+^ influx during reverse mode limiting Ca^2+^ overload and attenuates depolarization during forward mode reducing triggered activity (Antoons et al., 2012). However, if NCX1 is already diminished as shown in many models of diabetes (Makino et al., 1987; Pierce et al., 1990; Golfman et al., 1998; Hattori et al., 2000) additional inhibition was proven to be detrimental leading to adverse accumulation of Ca^2+^ in cytosol and cell death (Bögeholz et al., 2017).

### Targeting SERCa2a

SERCa2a overexpression and/or enhancement may be a more suitable approach to ameliorate reduced contractility in diabetic cardiomyopathy, given the majority of studies report diminished pump activity. Insulin treatment has been shown to restore SERCa2a expression levels and improve Ca^2+^ homeostasis in obese type 2 diabetic rats (Fredersdorf et al., 2012). Transgenic overexpression of SERCa2a (or SERCa1a) in diabetic models increased SR Ca^2+^ uptake and attenuated diminished contractile function (Trost et al., 2002; Vetter et al., 2002; Waller et al., 2015). As previously discussed, studies using adenoviral mediated SERCa2a gene transfer in both small and large animal models of HF demonstrated similar results (del Monte et al., 2001, 2004; Lyon et al., 2011). While gene transfer via adeno-associated virus showed promise in the first human trial, it has shown limited success in others. Improvements and advances in gene therapy technology are likely to facilitate efficient and effective strategies to treat cardiac disease in the future (Ishikawa and Hajjar, 2017).

To enhance SERCa2a activity one could also modulate the inhibitory action of the accessory protein PLB. Ablation or knockdown of PLB has been demonstrated to suppress pro-arrhythmic Ca^2+^ waves generation in a model of CPVT (Bai et al., 2013), improve mortality rates in CSQ-transgenic mice [severe HF model, (Kaneko et al., 2016)] and importantly, improve contractile function in failing human cardiomyocytes (del Monte et al., 2002). However, ablation has not alleviated HF development in all models and a mutant form of PLB unable to inhibit SERCa has been linked to lethal dilated cardiomyopathy in humans (Haghighi et al., 2003; Song et al., 2003; Sipido and Vangheluwe, 2010; Zhang et al., 2010).

### Targeting RyR2

Abnormally high activity of RyR2 is the most universal finding demonstrated across several models of diabetes. Compounds thought to modulate RyR2 including JTV-519 (K201), carvedilol, dantrolene and tetracaine analogs have previously been tested for therapeutic potential (Kaneko et al., 1997; Wehrens et al., 2004a; Kobayashi et al., 2010; Zhou et al., 2011; Zhang et al., 2015). However, there remains a need for drugs without off-target effects and significant effort is currently being made to identify small novel modulators of the channel that will prevent arrhythmogenic Ca^2+^ leak (Li et al., 2017; Rebbeck et al., 2017).

Stabilization of RyR2-mediated Ca release can be achieved indirectly by targeting CaMKII, given chronic activity in cardiac disease has been linked to RyR2 channel dysfunction (Ai et al., 2005; Uchinoumi et al., 2016; Zhang, 2017). It has been demonstrated that blockade of CaMKII and inhibition of RyR2 phosphorylation in cardiac disease improves intracellular Ca^2+^ homeostasis and attenuates arrhythmogenesis (Ather et al., 2013; Tzimas et al., 2015; Uchinoumi et al., 2016), including in a rat model of type 2 diabetes (Sommese et al., 2016), but this is also not a universal finding (Chakraborty et al., 2014). Alternatively, stabilization could be achieved by a reduction of RyR2 oxidation. In mouse and rat models of type 2 diabetes, it was demonstrated that treatment with ROS scavengers protects against spontaneous Ca^2+^ release events, blunting diastolic dysfunction and arrhythmogenesis *in vivo* (Shao et al., 2011; Joseph et al., 2016; Sommese et al., 2016; Sánchez et al., 2018). Antioxidants may also reduce ROS-dependent CaMKII activation, hence reduce RyR2 phosphorylation and elevated SR Ca^2+^ leak (Luczak and Anderson, 2014; Sommese et al., 2016; Uchinoumi et al., 2016). While increased oxidative stress and ROS concentrations are a hallmark of multiple cardiac disease states including diabetes, usage of ROS scavengers as a therapeutic strategy is not straightforward because certain levels of intracellular ROS are essential for many physiological processes and the ability to target antioxidants to specific subcellular compartments remains limited (Zima et al., 2014; Dietl and Maack, 2017).

Targeting accessory proteins of RyR2 also offers therapeutic potential. Adenoviral overexpression of sorcin in the hearts of diabetic mice improved contractile function and increased the Ca^2+^ transient amplitude of isolated rat cardiomyocytes (Suarez et al., 2004). In recent work of Liu et al. (2018) it was demonstrated that gene transfer of modified CaM prolonged refractoriness of RyR2-mediated SR Ca^2+^ release, abolishing ventricular arrhythmias observed in a mouse model of CPVT.

## Conclusion

In conclusion, changes in cardiac Ca^2+^ homeostasis vary across different models of diabetes and obesity. Diabetes is a progressive disease and therefore results from different laboratories using the same model can differ. Longitudinal studies are warranted to resolve the ongoing discrepancies. Although there are many similarities with HF, there are substantial differences in Ca^2+^ handling in diabetic cardiomyopathy so the treatment strategies could be different.

Abnormalities in Ca^2+^ cycling sufficient to increase arrhythmic potential appear very early during disease progression of diabetes. The most consistent finding even at very early stages of disease is enhanced RyR2 activity, making it an attractive therapeutic target. Future studies are needed to identify the most suitable approaches for RyR2 stabilization in diabetes. SERCa2a is also attractive target, especially at the later stages of disease progression and cardiomyopathy development. However, it remains unclear whether SERCa2a function is depressed at the early stages when arrhythmic risk is already high. The emerging concept of a mitochondria-SR microdomain and its potential as a therapeutic target may warrant investigation, given mitochondrial dysfunction is a well-established in diabetes. However, levels of mitochondrial ROS emission should be confirmed at different stages of disease, given work of some laboratories suggesting there may be an adaptive improvement in metabolism of fatty acids and thus a reduction in ROS emission. Furthermore, there remains a need for studies using larger animal models of type 2 diabetes with physiology more analogous to that of humans.

## Author Contributions

SH and DT obtained funding, conceived of, and wrote the manuscript.

## Conflict of Interest Statement

The authors declare that the research was conducted in the absence of any commercial or financial relationships that could be construed as a potential conflict of interest.
